# Effect on autonomic nervous activity of applying hot towels for 10 s to the back during bed baths

**DOI:** 10.1186/s40101-020-00245-7

**Published:** 2020-11-19

**Authors:** Inaho Shishido, Issei Konya, Rika Yano

**Affiliations:** 1 Graduate School of Health Sciences, Hokkaido University, Hokkaido, Japan; 2 Faculty of Health Sciences, Hokkaido University, Hokkaido, Japan

**Keywords:** Bed bath, Comfort care, Skin temperature, Autonomic nervous system, Relaxation

## Abstract

**Background:**

Bed baths are a daily nursing activity to maintain patients’ hygiene. Those may provide not only comfort but also relaxation. Notably, applying a hot towel to the skin for 10 s (AHT10s) during bed baths helped to reduce the risk of skin tears and provided comfort and warmth in previous studies. However, it is still unclear whether autonomic nervous system is affected by bed baths. Thus, this study investigated the effect on the autonomic nervous activity of applying hot towels for 10 s to the back during bed baths.

**Methods:**

This crossover study had 50 participants (25 men and women each; average age 22.2 ± 1.6 years; average body mass index 21.4 ± 2.2 kg/m^2^) who took bed baths with and without (control condition: CON) AHT10s on their back. Skin temperature, heart rate variability (HRV), and blood pressure (BP) were measured. Subjective evaluations and the State-Trait Anxiety Inventory in Japanese were also performed.

**Results:**

A significant interaction of time and bed bath type on skin surface temperature was observed (*p* < .001). Regarding the means of skin surface temperature at each measurement time point, those for AHT10s were significantly higher than those for CON. Although the total state-anxiety score significantly decreased in both the bed bath types after intervention, the mean values of comfort and warmth were higher for bed baths with AHT10s than for CON (*p* < .05) during bed baths; AHT10s was significantly higher in warmth than CON after 15 min (*p* = .032). The interaction and main effects of time on HRV and BP and that of bed bath type were not significant.

**Conclusion:**

Bed baths that involved AHT10s caused participants to maintain a higher skin temperature and warmer feeling than under the wiping-only condition; they also provided comfort during the interventions. However, the bed baths with AHT10s did not allow participants to reach a relaxed state; moreover, there was no change in autonomic nerve activity. This may be due to participants’ increased anxiety from skin exposure and the intervention being limited to one part of the body.

## Background

Skin health and integrity are essential in many ways for maintaining health [1]. Personal hygiene assistance, including bed baths, is one of many elements that contribute to maintaining skin integrity [2]. Therefore, bed bath is key nursing activity to maintain hygiene and to provide comfort for bedridden patients who are unable to perform self-care due to chronic or acute illness [3, 4]. Furthermore, the quality and efficiency of bed baths can have large impact on quality of life, health, and health care [5, 6].

A literature review found that disposable bed baths without soap and water performed significantly better than the bed bath with soap and water in terms of skin hydration [7], skin abnormalities [6], and nurse satisfaction [8, 9] which were studied. On the other hand, it was reported that there was no difference in the number of bacteria [10–13], cost, or severe skin lesions [6] depending on the bed baths method. Regarding the time required for the bed baths, some studies report that disposable bed bath was shorter than bed bath with soap and water [9, 14], but others report that they were comparable [8]. Although evidences of bed baths are being constructed as mentioned above, in recent years, insufficient attention to patient satisfaction has been pointed out [3], despite the increasing focus on patient-centered care [15]. In fact, the evidence of the psychological reactions of the subjects was poor. It has reported that patients often feel uncomfortable and anxious during the bed baths with soap and water [12, 16, 17], and even with disposable bed baths without water and soap, some elderly patients still feel uncomfortable [6] and complain about improper cleaning duration and poor cleaning [14] Unfortunately, an interview survey to explore the bedridden patients’ experience with bed baths reported that the patients were not always asked their bed baths preferences and they believed that bath methods should be selected by care giver [4]. Suiting patients’ needs and preferences is patient-centered care, which is argued to contribute to patient satisfaction [15]. Therefore, Cowdell et al. [2] reported that future studies should include patient-reported outcomes, such as comfort and acceptability.

In Japan, nurses have reported that patients often find the application of hot towels to be “a warming and comfortable experience,” which is why, despite the lack of evidence, they routinely apply a hot towel during bed baths before wiping the patients’ skin. Shishido et al. [18] aimed to decide the optimal time of hot towel application. They found that 10 s was the ideal duration from increase of skin temperature and the height of subjective comfort during interventions. The bed baths that applied a hot towel for 10 s (AHT10s) increased skin temperature (+ 0.5 °C) and provided warmth and comfort. Furthermore, Shishido and Yano [19] performed bed baths with AHT10s to the forearms of elderly people. Although the stratum corneum water content increased, there was no change the transepidermal water loss after the bed baths following the AHT10s. These results indicated that the bed baths with AHT10s did not damage the skin’s barrier function. The bed baths with AHT10s also elicited comments such as “very pleasant” and “it is like taking a bath” [19, 20]. As elderly people exhibited a strong positive reaction in spite of the bed bath being restricted only to the forearm, we predicted that applying hot towels during bed baths even for a short 10-s duration could potentially cause relaxation.

Interventions with relaxation effects, such as footbaths and massages, reduce patients’ anxiety, stress, and pain; therefore, they are regarded as an important form of care for enhancing well-being [21–24]. Similarly, interventions like bed baths with AHT10s, which provide thermal stimulation, could be a source of comfort to participants [24, 25]. From these results, it can be concluded that comfort and relaxation are related. A reduction in heart rate (HR) by itself considered a sign of relaxation, caused either by an increase in parasympathetic nervous system (PNS) activity or a decrease in sympathetic nervous system (SNS) activity [26]. Generally, a high-frequency (HF) component is a marker for pure cardiac PNS activity, while the low-frequency/high-frequency (LF/HF) ratio reflects SNS activity [27]. In this regard, massages lower LF/HF ratio [28, 29] and increase HF [30]. Furthermore, footbaths also reduce LF/HF [24].

In addition, thermal stimulus is received on the skin surface and is transmitted to the spinal cord, medulla oblongata, midbrain, and cerebral cortex. It activates the activities of the insular cortex and amygdala, areas related to emotion processing [31], while also affecting the autonomic nervous system (ANS) and the prefrontal cortex. Therefore, bed baths with AHT10s providing warmth and comfort might affect the ANS, and might also induce a relaxation effect. However, no previous studies have verified this.

We considered that the back was the most effective site for AHT10s. The percentage of calcitonin gene-related peptide positive neurons, which have a sensory role, among total neurons projecting to the trapezius muscle was high [32]. Additionally, the large stimulus area may help contribute to creating a feeling of warmth [33]. In this study, therefore, it was considered that the effect by the thermal stimulation was strong on the back from the anatomical features, and we selected the back to apply position. Therefore, this study aimed to examine the effect on the autonomic nervous activity of applying hot towels for 10 s to the back during bed baths. We hypothesized that bed baths with AHT10s would decrease the LF/HF ratio and increase HF. We anticipated that performing the bed baths with AHT10s on subjects with a high level of stress, anxiety, or tension could maintain skin cleanliness, while also providing relaxation and alleviating these conditions.

## Methods

### Study design

The study was conducted between December 2019 and February 2020. The study used a crossover design. As a result, each of the participants received two different types of bed baths: (1) bed baths that incorporated applying hot towels for 10 s on the back before the skin was wiped (bed baths with AHT10s), and (2) bed baths that that incorporated applying dry towels for 10 s (control condition: CON). Half of the participants received the bed bath with AHT10s the first day, and another half with CON the first day. On day 2, the type of bed bath was switched each group. They were randomly allocated to a group in which the first type of bed bath preceded the second type and vice versa for the other group, taking into account the counterbalance.

### Participants

Participants included 50 healthy volunteers (> 20 years old) with no experience or knowledge of bed baths. Eligible participants were recruited from a national university via posters and flyers and all individuals agreed to participate in the study. They consisted of 25 men (50.0%) and 25 women (50.0%) and had a mean age of 22.2 ± 1.6 (Table 1). In each experiment, participants were encouraged to get enough sleep on the night before the experiment, and to lay off alcohol 8 h before the experiment. On the day of the experiment, participants were asked to refrain from eating spicy food or drinking large quantities of caffeinated beverages, performing strenuous exercises that might cause sweating, and eating at least 1 h before the experiment. The sample size was calculated using G*Power software (ver. 3.1.9) [34]. There was no prior literature that measured heart rate variability (HRV) during bed baths. Therefore, the sample size was calculated using the effect size of previous studies that measured HRV in other nursing care [28, 30] and similar interventions [18]. As a result, the one with the largest number of samples was used. Forty-five participants were calculated as the minimum sample size with the following settings: paired *t* test analysis, .05; power of test, .80; effect size, .43 [18]. Furthermore, considering the possibility of a 10% of the dropout rate, 50 participants were targeted.

**Table 1:** Subjective characteristics and implementation environment (*n* = 50)

	Mean (SD)		95% CI for the difference	*t* value	*P* value
	Bed bath with AHT10s	CON			
Age (years)	22.2 (1.6)		–		
Body mass index (kg/m^2^)	21.4 (2.2)		–		
Muscle mass (kg)	43.4 (8.0)		–		
Body fat percentage (%)	21.4 (8.1)		–		
Area of back (cm^2^)	1641.1 (253.3)		–		
Time required for bed bath	84.0 (4.0)	84.7 (3.9)	− 1.5–0.3	− 1.37	.176
Implementation environment					
Room temperature (°C)	25.7 (0.4)	25.6 (0.4)	− 1.4–1.0	− 0.34	.738
Humidity (%)	35.1 (3.3)	34.7 (3.4)	− 0.1–0.1	0.48	.634

Paired *t* test. The area of the back was calculated by measuring the length (transverse boundary line: both axillary lines, upper boundary line: line connecting both acromion, lower boundary line: line connecting both iliac crests). *AHT10s* applying hot towels for 10 s to the skin, *CI* confidence interval, *CON* the bed bath with applying dry towel to the skin, *SD* standard deviation

### Experiment procedure

The trials occurred within a 6-h window (10:00–16:00) when the ANS is considered stable [35]. Subjects received the two types of bed baths at the same time on another day. The experiment was conducted in a soundproof room which reduced noise to 35 dB, to block outside noise and artifacts. The temperature (24–26 °C) and humidity (40–60%) were controlled in this room. Based on the Japanese Industrial Standards Committee [36], which requires the illumination within a hospital room be at least 100 lx, the illumination was controlled to a value between 185 and 215 lx. The same researcher applied the hot towels and wiped the skin for each participant. Significant differences were not between conditions in the experimental environments during the bed baths (Table 1).

### Intervention

In order to reduce the initial effect, subjects watched a video of the bed bath being performed before the experiment. Participants changed the hospital gown and were then placed in a sitting position, leaning forward to lightly hug a pillow placed on table.

#### Bed baths with AHT10s

**Fig. 1:**
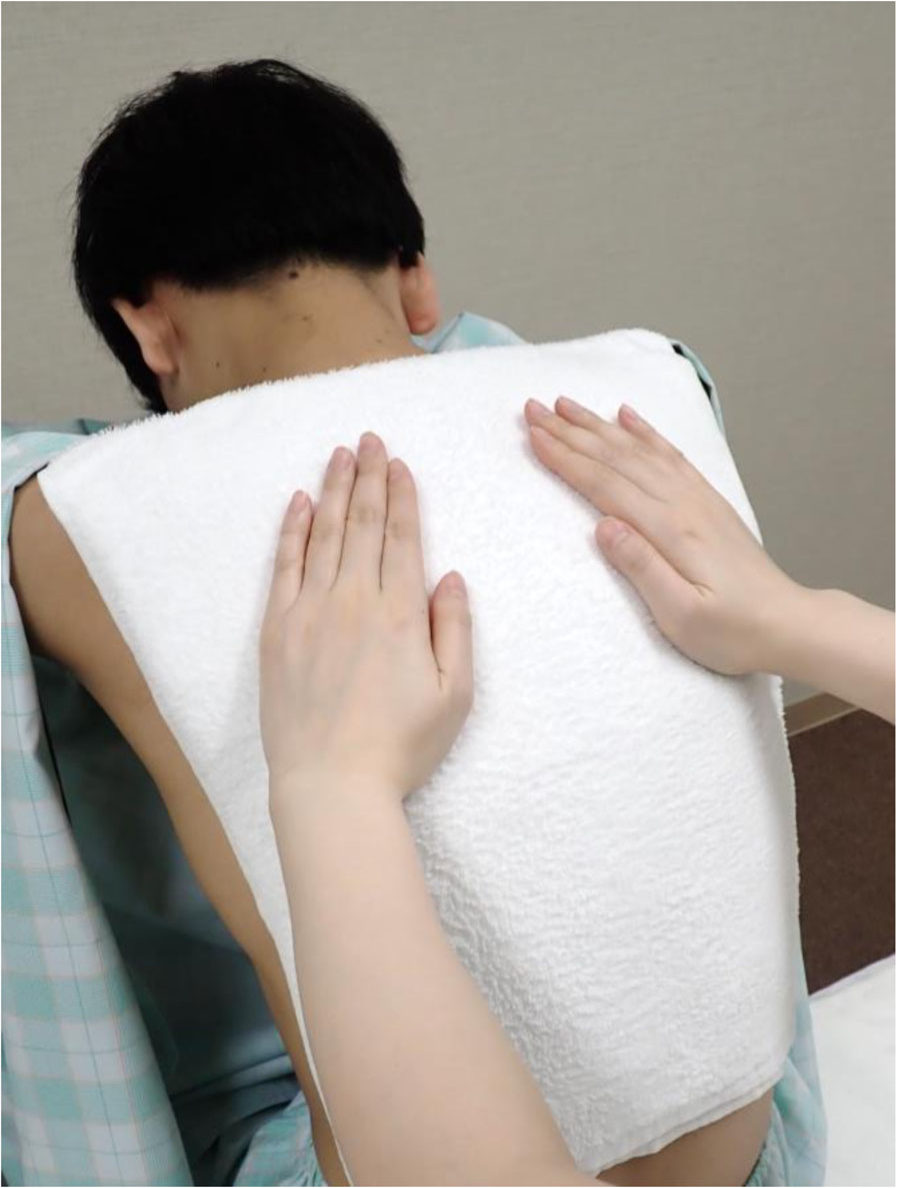
Application of the hot towels to the back. The hot towels applied to participants’ back, with the upper edge of the towels along placed a line connecting the right and left acromion. The researcher’s hands were placed on the hot towel, carefully applying constant pressure from the center of the towel

**Fig. 2:**
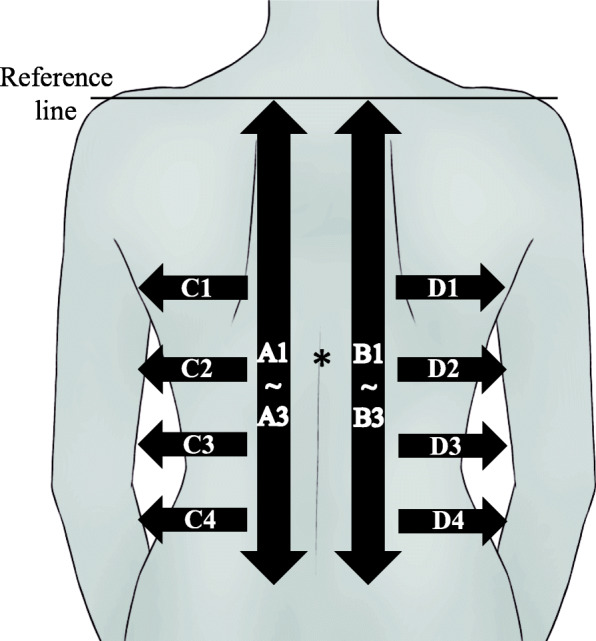
Experimental setup. Reference line, a line connecting the right and left acromion. * The skin temperature of the back was measured attaching a probe. Wiping was performed in the direction of the arrow in the time series shown

The time of the hot towel application was 10 s in this study. This is because in a previous study [18], the evaluation of comfort was found to be the highest when a hot towel was applied for 10 s, with some people complaining of coldness or discomfort when the towel was applied for 15 s or more. The protocol of the bed bathing methods is shown below. Two cotton hand towels (74 × 35 cm, 82.0 ± 2.0 g) were placed in a thermostatically controlled bath (TR-2AR Thermal Robo; As One, Osaka, Japan) filled with water at a temperature of 50 ± 0.02 °C. After heating, excess water was wrung out of the towels, leaving them with the final weight of 480.0 ± 5.0 g. The hot towels folded in two were immediately applied to participants’ back, with the upper edge of the towels placed along a line connecting the right and left acromion. The researcher’s hands were placed on the hot towels, carefully applying constant pressure from the center of the towel; held in that state for 10 s (AHT10s) (Fig. 1). After removing the hot towels, the left and right backs were wiped three times each in a direction parallel to the spinal column (Fig. 2(A1–A3 and B1–B3)) with another hot towel (final weight of 240.0 ± 5.0 g). Furthermore, using the same hot towel, the left and right flanks were wiped four times in a direction perpendicular to the spinal column (C1–C4 and D1–D4). Then, the skin was wiped with a dry towel. The pressure for applying and wiping was 23–25 mmHg [37]. The hot towel surface was replaced with an unused surface every time one part was wiped. After completion of the bed bath, the subject was made to wear clothes immediately.

#### Bed baths with applying dry towels (CON)

The dry hand towels, which were not heated were folded in two and immediately applied to participants’ back, with the upper edge of the towels placed along a line connecting the right and left acromion. The researcher’s hands were placed on the hot towels, carefully applying constant pressure from the center of the towels; held in that state for 10 s. After removal of the dry towels, wiping was performed in the same method as the baths with AHT10s.

### Data collection

**Fig. 3:**
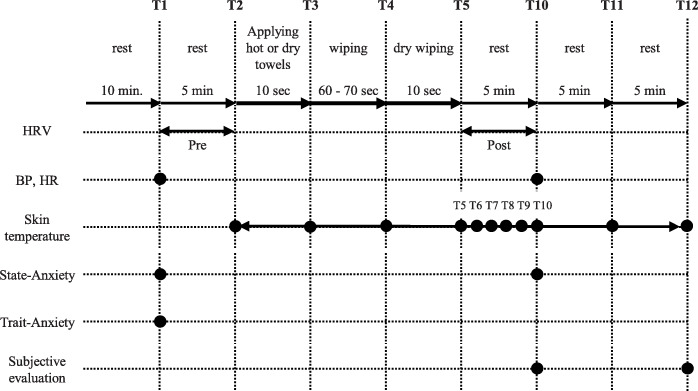
The experimental protocol. *BP* blood pressure, *HR* heart rate, *HRV* heart rate variability. T1, 5 min before intervention: T2, the beginning of the experiment: T3, immediately after applying the hot or dry towel to the skin: T4, immediately after wiping the skin: T5, immediately after wiping the skin with the dry towel: T6–10, 1–5 min after T5: T11, 10 min after T5: T12, 15 min after T5. Pre, between T1 and T2: Post, between T5 and T10

The total time for this trial was approximately 32 min. Data were collected 5 min before intervention (T1), at the beginning of the experiment (T2), immediately after applying the hot towels to the skin (T3), immediately after wiping the skin (T4), immediately after wiping the skin with the dry towel (T5), 1–5 min after T5 (T6–10), 10 min after T5 (T11), and 15 min after T5 (T12) (Fig. 3). “Pre” was defined as the period between T1 and T2, and “Post” between T6 and T10.

### Physiological measurements

#### Heart rate variability

An electrocardiogram (ECG) was performed with electrodes (Vitrode F-150M; Nihon kohden, Tokyo, Japan) attached to the chest. The ECG data was monitored by using a data logger (Biolog, DL-2000; S&ME Inc, Tokyo, Japan), HRV sensor (DL-310; S&ME Inc). All participants were asked to sit comfortably on a bed for 10 min before the recording of any ECG data. Baseline data were recorded for 5 min followed by intervention. During the intervention period, all subjects remained seated on the same bed that they were on before ECG data was being recorded. After the intervention period, all subjects remained seated while ECG data was recorded for 5 min. The HR and beat-to-beat detection (R-R interval) were gained using R-wave of the ECG. An HF component (0.15–0.40 Hz) and an LF component (0.04–0.15 Hz) of the power spectrum of R-R interval variability was determined by using the maximum entropy method (Memcalc/TARAWA; SUWA Trust, Tokyo, Japan). These values were used to determine the 5-min averages for HF component (ms^2^) reflecting the PNS activity for and LF/HF ratio reflecting the SNS activity [27].

#### Blood pressure

Blood pressure (BP) was measured with an electronic sphygmomanometer (ES-H56; Terumo, Tokyo, Japan) at T1 and T10.

#### Skin temperature on the back

Only the skin temperature was measured in this study because the bed baths were for a short duration (80 to 90 s), the thermal stimulus was small, and the possibility of changing the core temperature was low. The skin temperature of the back was measured by attaching a probe (Hardware N543; Nikkiso-Therm, Tokyo, Japan; accuracy, ± 0.1 °C) every second starting from before bed bath (T1) to 15 min after dry wiping (T11), and the value at which data was extracted at each time point was taken as the measured value (Fig. 3). We attached the probe at only a single location on the back: (1) to confirm whether an increase in skin temperature could be observed, as in previous research [18, 19]; (2) because the attached probe interferes with wiping; and (3) because the heat of the hot towel is not directly conducted to the skin by attaching probes at multiple locations.

### Psychological measurements

#### State-Trait Anxiety Inventory-JYZ

The State-Trait Anxiety Inventory-JYZ (STAI-JYZ) instrument was used to assess self-reported anxiety levels. The STAI-JYZ translated STAI-Y [38] into Japanese to reflect the cultural background of Japan and traits of Japanese people [39]. It can measure both “trait-anxiety” and “state-anxiety”. Trait-anxiety shows a tendency for an individual perception or response to a threatening situation, and state-anxiety shows transient response to an anxiety-provoking event. Participants answered questions about state-anxiety before and after the bed baths, and questions about trait-anxiety only before the bed baths. The inventory consisted of 20 trait and 20 state-anxiety items and were summed for a total score ranging from 20 to 80, with scores based on a four-point Likert scale (1, Not at all; 2, Somewhat; 3, Moderately so; 4, Very much so), with higher scores reflecting higher levels of anxiety. Hidano et al. [39] provided reference values for STAI-JYZ measurements (High anxiety: 55–80; Low anxiety: 20–44).

#### Subjective evaluation

Questionnaires were administered to participants following each bed bath type at T10 and T12. At T10, the subject confirmed the sensation during bed bathing. Each participant rated comfort, warmth, ability to remove dirt, and sleepiness on a five-point Likert scale (1, Not at all; 2, Not so much; 3, Neither; 4, Somewhat so; 5, Very much so). At T12, 15 min after the end of wiping, each participant then rated the comfort, warmth, and sleepiness on the same five-point Likert scale.

### Data analysis

HRV, BP, skin temperature, and state-anxiety were analyzed using mixed-linear model for two-way repeated-measures ANOVA, and a Bonferroni correction was carried out to adjust for multiplicity. In the mixed-linear ANOVA model, time and types were defined as a fixed factor, while subjects were defined as a random factor. The factors in this ANOVA were “time” (pre and post or T2–T12) and “bed bath type” (bed bath with AHT10s or CON). For the interaction, “time and bed bath type” was set up. If at least one of the factors or interactions was significant, single-factor repeated-measures ANOVA and multiple comparison tests were performed on the means. Paired *t* tests were also performed on the means to compare data between treatment types. Analyses were conducted using SPSS version 26 statistical software (IBM, Armonk, New York, NY, USA).

### Ethical considerations

People interested in participating were given verbal and written explanations about the following points: the study objective, experimental methods, the right to dropout at any time, and assurance that anonymity would be protected. All participants provided informed consent. If a participant grumbled of excessive heat or pain, the experiment was immediately stopped and the skin cooled. This study was approved by the Ethical Review Board of the authors’ affiliated university (approval no.19-68) and was performed in accordance with the Declaration of Helsinki.

## Results

### HRV

Forty-three subjects (22 men and 21 women), excluding seven subjects whose HRV values were more than 1.5 times the 1st and 3rd quartiles of the interquartile range, were analyzed. Nunan et al. [40] found that the mean of the HF component was 657 ± 777 ms^2^ and that of LF/HF ratio was 2.8 ± 2.6 in healthy adults. The main effects of time on HF component and LF/HF ratio, and that of bed bath type were not significant (Table 2). The interactions did not show any significance.

**Table 2:** Comparison of bed baths with AHT10s and CON: HRV, HR, BP (*n* = 43)

	Bed bath with AHT10s		CON		Main effect				Interaction type х time	
					Type		Time			
	Pre	Post	Pre	Post	*F* (*df*)	*P*	*F* (*df*)	*P*	*F* (*df*)	*P*
HF (ms^2^)										
Mean (SD)	567.3 (326.6)	569.3 (345.7)	536.7 (361.7)	600.8 (459.8)	< 0.01 (1,126)	.990	0.87 (1,126)	.353	0.77 (1,126)	.383
95% CI	462.9–675.7	462.9–675.7	425.4–648.0	459.3–742.3						
LF/HF										
Mean (SD)	1.9 (1.5)	2.6 (2.3)	2.5 (1.8)	2.5 (1.9)	1.38 (1,126)	.243	3.33 (1,126)	.070	1.91 (1,126)	.170
95% CI	1.5–2.4	1.9–3.3	1.9–3.0	2.0–3.1						
HR (bpm)										
Mean (SD)	68.9 (10.5)	68.3 (8.8)	68.3 (9.8)	68.0 (10.4)	1.05 (1, 126)	.308	0.51 (1, 126)	.475	0.07 (1, 126)	.785
95% CI	65.7–72.1	65.6–71.0	65.3–71.3	64.8–71.2						
SBP (mmHg)										
Mean (SD)	106.4 (9.6)	107.4 (10.3)	106.7 (10.3)	105.6 (11.5)	0.56 (1, 126)	.457	< 0.01 (1, 126)	.968	0.93 (1, 126)	.337
95% CI	103.5–109.4	104.3–110.6	103.5–109.9	102.0–109.1						
DBP (mmHg)										
Mean (SD)	63.7 (7.1)	62.9 (7.8)	64.0 (8.0)	63.8 (7.4)	1.23 (1, 126)	.271	0.83 (1, 126)	.365	0.14 (1, 126)	.705
95% CI	61.5–65.8	60.5–65.3	61.6–66.5	61.6–66.1						

A mixed-linear model for two-way repeated-measures ANOVA, and a Bonferroni correction. *AHT10s* applying hot towels for 10 s to the skin, *BP* blood pressure, *CI* confidence interval, *CON* the bed bath with applying dry towel to the skin, *DBP* diastolic blood pressure, *LF* low frequency component (0.04–0.15 Hz), *HF* high-frequency component (0.15–0.40 Hz), *HR* heart rate, *HRV* heart rate variability, *SD* standard deviation, *SBP* systolic blood pressure. Pre, between T1 (5 min before intervention) and T2 (the beginning of the experiment): Post, between T6 (immediately after wiping the skin with the dry towel) and T10 (5 min after wiping the skin with the dry towel)

### HR, systolic blood pressure, and diastolic blood pressure

HR, systolic blood pressure (SBP), and diastolic blood pressure (DBP) values did not show a significant main effect for time and bed bath type. The interactions were not significant (Table 2).

### Skin temperature on the back

**Fig. 4:**
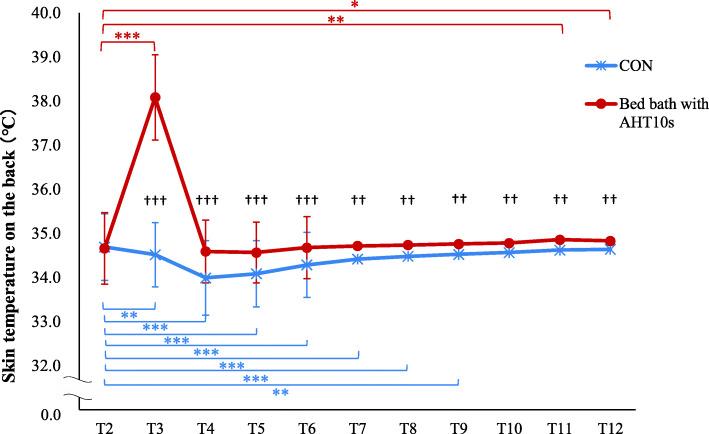
Time courses of skin temperature of healthy adults (*n* = 50). † Paired *t* test. †††*p* < .001, ††*p* < .01. * Mixed-linear model for two-way repeated-measures ANOVA, and a Bonferroni correction. ***p < .001, **p < .01, *p < .05. AHT10s, applying hot towels for 10 s to the skin: CON, the bed bath with applying dry towel to the skin. T2, the beginning of the experiment: T3, immediately after applying the hot or dry towel to the skin: T4, immediately after wiping the skin: T5, immediately after wiping the skin with the dry towel: T6–10, 1–5 min after T5: T11, 10 min after T5: T12, 15 min after T5

The mean skin temperature on the back at T2 was 34.7 ± 0.8 (bed baths with AHT10s) or 34.7 ± 0.7 °C (CON). There were significant main effects of time on skin temperature [*F* (10,1029) = 257.64, *p* < .001] and bed bath type [*F* (1,1029) = 815.27, *p* < .001]. A significant interaction of time and bed bath type was observed [*F* (10,1029) = 219.90, *p* < .001] (Fig. 4). For the mean at each measurement point, those for AHT10s were significantly higher than those for CON at T3 to T12 (*p* < .01). The mean skin temperature was significantly higher at T3 (3.4 ± 0.8 °C, *p* < .001), T11 (0.2 ± 0.4 °C, *p* = .002), and T12 (0.2 ± 0.4 °C, *p* = .016) relative to T2 for bed baths involving AHT10s. However, the mean skin temperature at T3 to T10 was significantly lower than at T2 (*p* < .05) for CON.

### STAI-JYZ

The score of trait-anxiety for the subjects in this study was slightly lower but still similar to the average value for healthy adults [39]. Although there was a significant main effect of time on state-anxiety, was not of bed bath type (Table 3). The mean of state-anxiety score of both types of bed bath decreased significantly at T10 (*p* < .001). There were no significant differences between the two types of bed baths in trait-anxiety before the experiment.

**Table 3:** Comparison of bed baths with AHT10s and CON: STAI (*n* = 50)

		Bed bath with AHT10s		CON		Main effect				InteractionType х Time	
						Type		Time			
		Mean (SD)	95% CI	Mean (SD)	95% CI	*F* (*df*)	*P*	*F* (*df*)	*P*	*F* (*df*)	*P*
State-anxiety	Pre	36.3 (7.6)	34.1–38.5	35.7 (6.5)	33.9–37.6	0.02 (1, 147)	.853	51.54 (1, 147)	<.001	1.08 (1, 147)	.301
	Post	32.2 (6.3)***	30.4–34.0	32.7 (5.7)***	31.1–34.3						
Trait-anxiety		45.4 (10.0)	42.6–48.3	44.8 (9.9)	42.0–47.6	–	–	–	–	–	–

A mixed-linear model for two-way repeated-measures ANOVA, and a Bonferroni correction. ***p < .001. *AHT10s* applying hot towels for 10 s to the skin, *CI* confidence interval, *CON* the bed bath with applying dry towel to the skin, *SD* standard deviation. Pre, between T1 (5 min before intervention) and T2 (the beginning of the experiment): Post, between T6 (immediately after wiping the skin with the dry towel) and T10 (5 min after wiping the skin with the dry towel)

### Subjective evaluation

The mean values for comfort and warmth at T10 of bed baths with AHT10s were significantly higher than those of CON (*p* < .05) (Table 4). At T12, AHT10s was significantly higher in warmth than CON (*p* = .032).

**Table 4:** Comparison of bed baths with AHT10s and CON: Subjective evaluation (*n* = 50)

	Mean (SD)		95% CI for the difference	*t* value	*P* value
	Bed bath with AHT10s	CON			
5 min after dry wiping (T10)					
Comfort	4.6 (0.6)	4.4 (0.7)	0.0–0.4	2.48	.017
Warmth	4.8 (0.4)	4.2 (0.9)	0.3–0.8	4.60	< .001
Ability to remove dirt	3.9 (0.8)	3.7 (0.8)	− 0.0–0.4	1.70	.095
Sleepiness	3.3 (1.2)	3.4 (1.1)	− 0.5–0.3	− 0.57	.569
15 min after dry wiping (T12)					
Comfort	4.1 (0.6)	3.9 (0.8)	− 0.0–0.4	1.59	.118
Warmth	3.6 (0.9)	3.3 (1.0)	0.4–1.0	2.64	.011
Sleepiness	3.8 (1.1)	3.7 (1.0)	0.1–1.1	0.39	.700

Paired *t* test. *AHT10s* applying hot towels for 10 s to the skin, *CI* confidence interval, *CON* the bed bath with applying dry towel to the skin, *SD* standard deviation

## Discussion

In this study, we investigated whether the warmth and comfort of bed baths using the hot towels application to the back affected ANS in 50 healthy adults. Our results showed that, following a bed bath with AHT10s on the back, skin temperature increased (+ 3.4 °C), as in a previous study [18], and was significantly higher than that of CON until 15 min after bed baths. In addition, bed baths with AHT10s provided warmth until 15 min after bed baths, and both warmth and comfort were significantly higher for the bed baths with AHT10s than for CON at 5 min after the end of dry wiping. However, HRV, which is an index of ANS, and HR and BP, which are related indicators, did not change after the bed baths, and there was no significant difference between the bed baths with AHT10s and CON conditions. These results suggested that short-term warmth and comfort did not necessarily lead to a relaxation effect. This result has a similarity that is found by a previous research [41] in which a 10-min warm hand bath was administered; the results of subjective evaluation and HRV suggested that the intervention increased subjective warmth and comfort without affecting ANS.

There may be two reasons why AHT10s did not affect ANS. First, the intervention was limited to one part of the body. For stimulus reception and emotion, cutaneous C-tactile afferents send a signal to areas of the brain involved with positive emotions [42]. In other words, the stimulation of C-tactile afferents could be related to the feeling of comfort, and continuous warm stimulation of skin with C-tactile afferents might lead to a continued sense of comfort. This suggests that in Kudo and Sasaki’s [41] study and our study, because the intervention was applied to only one part of the body, the skin stimulation was not strong enough to influence ANS, resulting in no relaxation effect. However, we did not examine whole-body effects to avoid influencing HRV from changes in posture and the burden associated with prolonged rest. Thus, the relaxation effect and the maintenance of comfort and warmth due to AHT10s to the whole body should be verified in future studies.

The second reason relates to the possibility of increased anxiety associated with skin exposure. Although bed baths constitute a fundamental form of care in maintaining patients’ skin hygiene, they could expose patients’ skin to others and be considered an invasion of privacy [17]. It has been reported that a lack of this awareness among professionals causes anxiety in patients, and their anxiety during bed baths was stronger than that during shower baths [16]. The mean of the state-anxiety score for both types of bed baths decreased significantly in this study; however, it may have been difficult for subjects to fully relax during the bed baths because of exposed skin.

Contrary to the hypothesis, the LF/HF ratio for bed baths with AHT10s tended to increase after bed bathing, although there was no significant difference between the before and after values, and thermal stimulation might have activated the SNS. In addition to increasing subjective comfort, thermal stimulation activates brain areas such as the orbitofrontal, medial prefrontal, and cingulate cortices [43, 44]. The orbitofrontal cortex and medial prefrontal cortex are primarily behavior-related [45] and play an important role in intrinsic motivation, such as working hard for your own benefit [46]. Furthermore, Kolcaba [47] reported that when comfort is enhanced, individuals are motivated to engage in health-seeking behaviors, with an ultimate improvement in outcome. Based on these results, future studies should evaluate the effect of bed baths while applying hot towels on the back from multiple aspects, such as the cognitive-behavioral aspect, and by mapping brain activity rather than assessing the effect only on the ANS.

## Conclusions

Since the heat from the hot towel was transferred to the skin, the bed baths that involved AHT10s caused participants to maintain a higher skin temperature and warmer feeling than under the wiping-only condition and also provided comfort during the intervention. Though an effect on the ANS from brain region activity (for example, activity in the hypothalamus from thermal stimulation) has been reported in preceding research, the bed baths with AHT10s did not affect the ANS; therefore, a relaxation effect might not have been achieved, even if warmth and comfort were obtained. This may be due to participants’ increased anxiety from skin exposure and that the intervention was limited to one part of the body. Further, the results of previous studies on the effects of nursing care on brain region activity and comfort in response to stimulation indicate that the LF/HF ratio tended to increase for the bed baths with AHT10s. Therefore, it is expected that comfort will impact a person’s cognitive behavior, so it is necessary to further verify the effect of bed baths with AHT10s.

## Data Availability

The datasets during and/ or analyzed the current study are available from the corresponding author on reasonable request.

## Abbreviations

AHT10s: Applying hot towels for 10 s to the skin
ANS: Autonomic nervous system
BP: Blood pressure
CON: Bed baths with applying dry towels for 10 s to the skin
DBP: Diastolic blood pressure
ECG: Electrocardiogram
LF: Low-frequency
HF: High-frequency
HR: Heart rate
HRV: Heart rate variability
PNS: Parasympathetic nervous system
SBP: Systolic blood pressure
SNS: Sympathetic nervous system
STAI: State-Trait Anxiety Inventory
